# Of Monkeys and Men: Immunomic Profiling of Sera from Humans and Non-Human Primates Resistant to Schistosomiasis Reveals Novel Potential Vaccine Candidates

**DOI:** 10.3389/fimmu.2015.00213

**Published:** 2015-05-05

**Authors:** Mark S. Pearson, Luke Becker, Patrick Driguez, Neil D. Young, Soraya Gaze, Tiago Mendes, Xiao-Hong Li, Denise L. Doolan, Nicholas Midzi, Takafira Mduluza, Donald P. McManus, R. Alan Wilson, Jeffrey M. Bethony, Norman Nausch, Francisca Mutapi, Philip L. Felgner, Alex Loukas

**Affiliations:** ^1^Centre for Biodiscovery and Molecular Development of Therapeutics, Australian Institute of Tropical Health and Medicine, James Cook University, Cairns, QLD, Australia; ^2^QIMR Berghofer Medical Research Institute, Brisbane, QLD, Australia; ^3^University of Melbourne, Melbourne, VIC, Australia; ^4^Centro de Pesquisas Rene Rachou, Oswaldo Cruz Foundation, Belo Horizonte, Brazil; ^5^Federal University of Minas Gerais, Belo Horizonte, Brazil; ^6^National Institute of Parasitic Diseases, Shanghai, China; ^7^National Institutes of Health Research, Harare, Zimbabwe; ^8^Department of Biochemistry, University of Zimbabwe, Harare, Zimbabwe; ^9^Department of Biology, University of York, York, UK; ^10^Department of Microbiology, Immunology and Tropical Medicine, George Washington University, Washington, DC, USA; ^11^University of Edinburgh, Edinburgh, UK; ^12^University of California Irvine, Irvine, CA, USA

**Keywords:** schistosomiasis, protein microarray, vaccine, human, drug-induced resistance

## Abstract

*Schistosoma haematobium* affects more than 100 million people throughout Africa and is the causative agent of urogenital schistosomiasis. The parasite is strongly associated with urothelial cancer in infected individuals and as such is designated a group I carcinogen by the International Agency for Research on Cancer. Using a protein microarray containing schistosome proteins, we sought to identify antigens that were the targets of protective IgG1 immune responses in *S. haematobium*-exposed individuals that acquire drug-induced resistance (DIR) to schistosomiasis after praziquantel treatment. Numerous antigens with known vaccine potential were identified, including calpain (Smp80), tetraspanins, glutathione-*S*-transferases, and glucose transporters (SGTP1), as well as previously uncharacterized proteins. Reactive IgG1 responses were not elevated in exposed individuals who did not acquire DIR. To complement our human subjects study, we screened for antigen targets of rhesus macaques rendered resistant to *S. japonicum* by experimental infection followed by self-cure, and discovered a number of new and known vaccine targets, including major targets recognized by our human subjects. This study has further validated the immunomics-based approach to schistosomiasis vaccine antigen discovery and identified numerous novel potential vaccine antigens.

## Introduction

The carcinogenic blood fluke, *Schistosoma haematobium*, infects more than 100 million people throughout Africa and is the most prevalent of the human schistosomes, causing more than half of all infections (1). *S. haematobium* adult flukes migrate to the vasculature of the organs of the pelvis. Severe morbidity results from host immune responses to eggs in tissues and includes periportal fibrosis, portal hypertension, and hepato-splenic disease (2). Formerly known as urinary schistosomiasis, *S. haematobium* infection was recently renamed “urogenital schistosomiasis” in recognition that the disease affects both the urinary and genital tracts of women and men. Female *S. haematobium* lay between 20 and 200 eggs daily (3), which penetrate the vessel wall and move toward the lumen of the bladder. Some of the eggs become sequestered in the tissue of the pelvic organs such as the urinary bladder, ureters, cervix, vagina, prostate gland, and seminal vesicles, where they cause chronic inflammation, pelvic pain, bleeding, and an altered cervical epithelium in women (4). *S. haematobium* is unique among the schistosomes in its recognition as a group I carcinogen by the International Agency for Research on Cancer because of its robust association with urothelial carcinoma (5). *S. haematobium* infection also increases susceptibility to infection with HIV-1, progression to disease, and results in a higher likelihood of transmitting infection to others (6).

Praziquantel (PZQ) is widely used to treat human schistosome infections and has two main effects on schistosomes – paralysis and tegument damage (7). An added benefit of PZQ treatment is that it mediates destruction of flukes thereby exposing antigens on the worm surface to the host immune system. This release of surface antigens induces and/or enhances parasite-specific immune responses (8), resulting in immune-mediated killing of the parasite. Early studies reported modifications in T-cell proliferative responses (9), whereas recent studies noted modifications in the levels and types of antibody (10–13) and cytokine responses (14–16) following PZQ treatment. The immune response triggered by PZQ treatment is thought to last for more than 1 year (14, 17–19) and confer at least some level of resistance to re-infection. This phenomenon is referred to as “drug-induced resistance” (DIR) (20). The mechanisms behind DIR differ significantly from those of putative natural resistance (PR, resistant individuals who have not received PZQ therapy) and can be related to the origin (developmental stage) and concentration of the released antigen, as well as the type of antigen-presenting cells (APCs) involved. PZQ treatment introduces a large amount of adult fluke antigen directly into the bloodstream as a result of many worms dying at once (21), whereas naturally acquired resistance in the absence of PZQ treatment (PR) is stimulated by the introduction of smaller quantities of adult antigen due to a more gradual worm death. The process of PR is additionally stimulated by the release of antigens from naturally dying larval schistosomes (schistosomula) primarily through the skin and pulmonary vasculature, thus inducing different APCs and resulting in different interactions between the antigens and the immune system (22). This additional stimulus does not appear to factor significantly in DIR due to the ineffectiveness of PZQ against schistosomula (7, 8). Whatever the mechanism, it is important that an antigen threshold is reached in order to sufficiently stimulate anti-schistosome immunity (23, 24).

Studies with car washers in schistosome-infected waters of Lake Victoria in Kenya showed that a subset of the men developed resistance to re-infection after PZQ therapy while others remained susceptible despite treatment (25, 26). It was found that IgE production to soluble worm antigen preparation (SWAP) paralleled the development of resistance, and did not occur in those who remained susceptible to re-infection (25). Additionally, our own immuno-proteomic studies have used *S. haematobium* SWAP to identify a number of antigens that are released by PZQ treatment and/or are the target of DIR immune responses (27, 28). However, despite the power of these proteomic studies in identifying individual parasite proteins, the utilization of SWAP (where worms are homogenized and solubilized under native conditions in the absence of detergents that will solubilize the cell membranes) does not result in full representation of the *S. haematobium* proteome. Indeed, numerous abundantly expressed proteins with multiple membrane spanning domains that are released from the tegument with detergents (29, 30) are accessible to chemical labeling on the surface of live worms (30), are recognized by sera from PR individuals, and are lead vaccine antigens against schistosomiasis (31–33).

A third mechanism of resistance to schistosomiasis is seen in the rhesus macaque (*Macaca mulatta*). It is unique among animal models of schistosomiasis in that, once an infection reaches patency, worm death starts to occur from week 10 (34) and egg output diminishes over time until the infection is eliminated (35, 36). This phenomenon only occurs above a threshold worm burden (35, 36), presumably as sufficient immune stimulus is required for this process to occur (23, 24). This self-cure mechanism is thought to be antibody-mediated because of a strong inverse association between the rapidity and intensity of the IgG response and the number and morphology of surviving worms (34). Two-dimensional immunoblotting of worm extracts showed the immune response to be directed at gut digestive enzymes, tegument surface hydrolases, and anti-oxidant enzymes (34).

The use of protein microarrays to profile the immune response to pathogens has become widespread over recent years and offers significant advantages over the conventional immuno-proteomic approaches described above. In parasitology, protein array studies have been used extensively in malaria (37) to compare antibodies from un-protected and protected subjects, identifying the antibodies (and their cognate antigens) that confer immunity (38–40). For schistosomes (37), similar studies have profiled antibody responses in *S. japonicum*- and *S. mansoni*-infected rodents (41, 42) and human subjects who are naturally resistant or susceptible to *S. mansoni* (20).

Based on the success of our previous immunomics approach which analyzed antibody signatures of PR and chronically infected (CI) individuals from an *S. mansoni*-endemic area of Brazil (20), we decided to use the same experimental approach to identify antigens which are the targets of humoral immune responses in (1) DIR human subjects from an *S. haematobium-*endemic area in Africa and (2) rhesus macaques that had undergone self-cure after experimental *S. japonicum* infection. Given the extensive similarities in protein-coding gene sequences between the three major human schistosomes (86–92%) (43), as well as the extensive recognition of *S. japonicum* proteins on our array by sera from *S. mansoni*-infected individuals (20), we reasoned that sera from *S. haematobium*-infected individuals would strongly recognize many of the arrayed *S. mansoni* and *S. japonicum* proteins. Moreover, these cross-reactive antigens would potentially form the basis of a pan-schistosome vaccine that protects against all three human species. Leveraging existing protein arrays from our previous study, which contain antigens primarily from the antibody-accessible teguments of the adult fluke and the immunologically vulnerable schistosomulum stage, we show that DIR individuals and self-curing rhesus macaques make robust antibody responses to a number of tegument-associated proteins, including novel and previously described schistosome vaccine candidates.

## Materials and Methods

### Ethical statement

Ethical and institutional approval was granted by the Medical Research Council of Zimbabwe and the University of Zimbabwe Institutional Review Board. Local permission for the study was granted by the Provincial Medical Director. The study design, aims, and procedures were explained in the local language, Shona, prior to enrollment. Participants were free to drop out of the study at any time and informed written consent was obtained from all participants prior to taking part in the study and to receiving anthelmintic treatment. As routine, all participants were offered treatment with the standard dose of PZQ (40 mg/kg) at the end of the study. All work involving experimental procedures with Rhesus macaques was approved by the Ethics Committee of Kunming Institute of Zoology, Chinese Academy of Sciences (CAS) (ID: SYDW-2011017).

### Study cohort

**Figure 1:**
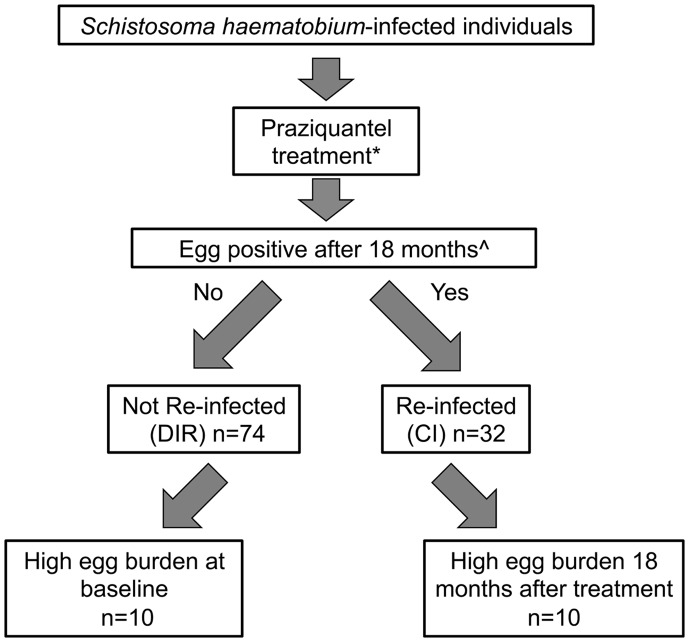
**Characterization of study cohort and sub-cohort used for the study described herein**. *Treatment efficacy was assessed by urinalysis 6 weeks after praziquantel therapy – all subjects were egg-negative (no eggs found in any of three urine samples, each collected on a separate day). ^∧^Subjects remained in the endemic study area and had regular water contact for the study duration.

The study participants were residents of a *S. haematobium*-endemic rural village in Murewa in the Mashonaland East Province of Zimbabwe (31°94′E; 17°67′S). The village was selected because health surveys regularly conducted in the region showed little or no infection with soil-transmitted helminths (STH) and a low *S. mansoni* prevalence (<2%). Serum samples were provided from a cohort of *S. haematobium*-infected individuals (*n* = 106) aged 5–14 years who had never been treated with PZQ prior to this study and were free from co-infection with other helminths, *Plasmodium*, and HIV (14, 44). At the start of the study (baseline), subjects who were positive for *S. haematobium* eggs (at least one egg found in at least one of three urine samples, each collected on a separate day) following urinalysis were treated with PZQ by weight (40 mg/kg) and then assessed by urinalysis at 6 weeks to confirm clearance of the infection (no eggs found in any of three urine samples, each collected on a separate day). Individuals were followed for 18 months and maintained regular water contact throughout this period. Subjects were assessed for infectivity with *S. haematobium* at 6 months and at the end of the study. Individuals who were egg-positive at 18 months post-treatment (*n* = 32) were deemed CI and those who were egg-negative (*n* = 74) were categorized as DIR (Figure 1). Serum samples were obtained from both 0- and 18-month timepoints.

For this study, we selected a subset of subjects as follows: CI subjects that had the highest post-treatment egg burdens (eggs/10 ml 10–104; *n* = 10) and DIR subjects that had some of the highest egg burdens at baseline (eggs/10 ml 44–743; *n* = 10), reasoning that these individuals represented extremities of the DIR and CI spectrums and therefore would maximize the likelihood of identifying differences in antibody signatures between CIs and DIRs. Subject ages (in years) were as follows: CIs (5, 8, 8, 9, 10, 10, 11, 11, 12, 14), range = 5–14, mean = 9.8, median = 10; DIRs (6, 8, 8, 8, 8, 9, 9, 10, 11, 12), range = 6–12, mean = 8.9, median = 8.5.

### Infection of self-curing rhesus macaques

The study used six captive-bred adult male rhesus macaques (*M. mulatta*; mean age 9.67 ± 0.82 years, mean weight 7.98 ± 0.85 kg) from the Kunming Primate Research Center, CAS. Macaques were group-housed prior to the experiment but then singly after infection for fecal sampling. Cercariae of *S. japonicum* were shed from patent snails (*Oncomelania hupensis*) provided by the Jiangsu Institute of Parasitic Diseases (Wuxi, China), collected from the water surface using a bacteriological loop and placed on glass cover slips for infection. Rhesus macaques anesthetized with ketamine hydrochloride (6 mg/kg body weight, Gutian Pharmaceutical Corporation, Fujian, China) were infected percutaneously with 600 cercariae via the shaved abdominal skin for 30 min. Blood was obtained by intravenous sampling prior to infection (week 0) and at 12 and 20 weeks after exposure. Elimination of infection was confirmed at week 20 by assessment of eggs per gram of feces using both the Percoll technique (45) and Kato-Katz method (46).

### Probing of protein microarrays with human and macaque sera

Protein microarrays were leveraged from a previous study by us (20) and contained both *S. mansoni* (*n* = 45) and *S. japonicum* (*n* = 172) proteins which were either (1) known or predicted to be localized to the tegument and/or (2) expressed in the schistosomulum (41), which is vulnerable to immune attack. Human IgG1 and IgE responses to antigens were determined by probing arrays with sera as previously described (20). Macaque antibody responses were determined by probing of arrays with sera as described for human sera with the exception that a goat anti-monkey IgG-biotin (1:500) (Sigma) secondary antibody was used.

### Protein array data analysis and bioinformatics

Array data analysis was conducted using the “group average” method (20), where the mean signal intensity (SI) of the negative control (empty vector) spots for all sera were subtracted from the SI of each protein spot. The following reactivity cut-offs (calculated as one standard deviation above the negative control spots for all groups) were used: human IgG1 – 8239; human IgE – 1861; macaque IgG – 3210. Statistical analyses (Student’s *t*-test) were conducted with Graphpad Prism 6 to determine significant differences between samples for a given reactive protein.

The transcription of genes in the adult and egg stages of *S. haematobium* was assessed for *S. haematobium* orthologs of all arrayed *S. mansoni* and *S. japonicum* proteins that were the targets of significantly different IgG responses between DIR and CI post-treatment sera using publicly available RNA-seq data (43). These data were filtered for quality (PHRED score of >30) using Trimmomatic (47) and aligned to the open reading frames of the published gene set (43) using Bowtie (v2.1.0) (48). Normalized levels of gene transcription were calculated using the software package RSEM (v1.2.11) (49) and reported as the numbers of transcripts per million reads sequenced (TPMs). The TPM value of each gene was log_2_-transformed and subjected to heat map visualization using R (v3.1.2)^1^, and utilizing the heatmap.plus (v1.3)^2^ package.

## Results

### Antibody signatures of DIR human subjects differ before and after PZQ treatment

**Figure 2:**
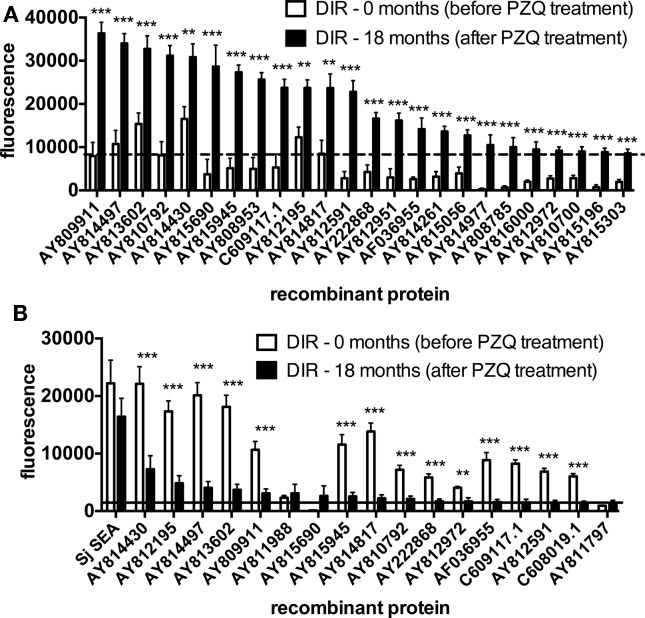
**Antibody responses to arrayed antigens differ in *Schistosoma haematobium*-infected humans before and after praziquantel treatment**. **(A)** IgG1. **(B)** IgE. Average adjusted signal intensity values depicting the antibody response to each reactive antigen are shown for the drug-induced resistant (DIR) cohort before and after praziquantel treatment. The dashed and solid lines are the respective cut-offs for IgG1 (8239) and IgE (1861) reactivity, calculated as one standard deviation of the mean of the no-DNA control spots on the array. Statistical analysis was performed using Student’s *t*-test. *p < 0.05, **p < 0.01, ***p < 0.001.

To investigate the difference in antibody responses to arrayed antigens of the DIR cohort before and after PZQ treatment (therefore identifying antigens which are putatively exposed by drug therapy), sera from this group at baseline and 18 months after drug therapy were used to probe protein microarrays. IgG1 responses were significantly higher in DIRs at 18 months post-treatment compared to baseline for all 24 reactive proteins. Antigens which were the target of the most significantly different (*p* < 0.0001) responses pre- and post-drug treatment included AY810700 (glucose transporter), AY815303 [glutathione-*S*-transferase (GST)], and AY809911 (Ig domain-containing, sensory guidance protein) (Figure 2A). In contrast, IgG1 responses of the CI cohort to reactive proteins before and after PZQ treatment were not significantly different for any protein (data not shown). Additionally, IgE responses in the DIR group were significantly lower at 18 months post-PZQ treatment compared to baseline for the majority (78%) of the 18 reactive antigens (Figure 2B). Arrayed antigens that were the targets of IgE in post-treatment DIRs included AY814430 (calpain), AY812195 [extracellular superoxide dismutase (SOD)], and AY814497 (Na^+^/K^+^ ATPase β subunit – SNaK1β).

### IgG1 profiles differ between *S. haematobium*-infected humans who do and do not acquire DIR after PZQ treatment

**Figure 3:**
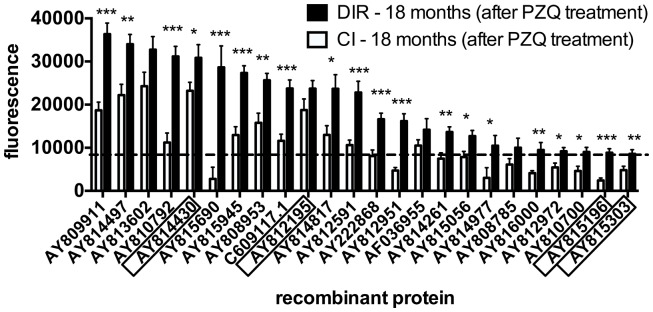
**IgG1 antibody profiles to arrayed antigens differ between *Schistosoma haematobium*-infected humans who do and do not acquire drug-induced resistance after praziquantel treatment**. Average adjusted signal intensity values depicting IgG1 antibody responses to each reactive antigen are shown for the drug-induced resistant (DIR) and chronically infected (CI) cohorts after praziquantel treatment. Boxed antigens indicate homologs of known vaccine candidates. The dashed line is the cut-off for IgG1 reactivity (8239), calculated as one standard deviation of the mean of the no-DNA control spots on the array. Statistical analysis was performed using Student’s *t*-test. *p < 0.05, **p < 0.01, ***p < 0.001.

**Figure 4:**
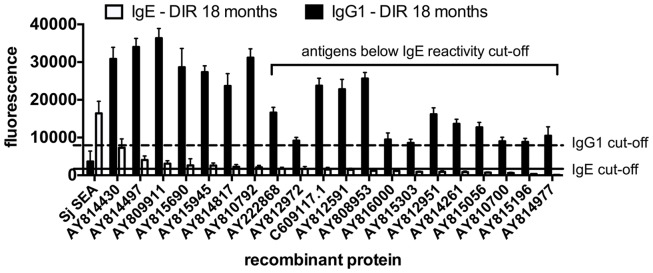
**Some arrayed antigens that induce IgG1 responses in *Schistosoma haematobium*-infected humans who acquire drug-induced resistance after praziquantel treatment are not the targets of IgE**. Average adjusted signal intensity values depicting IgG1 and IgE antibody responses to each IgG1 antigen reactive to post-treatment sera from drug-induced resistant (DIR) humans. The dashed and solid lines are the respective cut-offs for IgG1 (8239) and IgE (1861) reactivity, calculated as one standard deviation of the mean of the no-DNA control spots on the array. *Schistosoma japonicum* SEA is included for comparative purposes.

In order to analyze changes in antibody signatures to arrayed antigens related to the acquisition of DIR (thereby identifying proteins which are potential inducers of a protective antibody response), arrays were interrogated with sera from post-treatment CIs and DIRs and probed for IgG1 reactivity. IgG1 responses were significantly elevated in DIRs compared to CIs at 18 months to 20 of the 24 (83%) reactive proteins. The three antigens that were targets of the most significantly different (*p* < 0.0001) IgG1 responses were AY810792 (butylcholinesterase), AY812951 (mastin), and AY815196 [a homolog of human tetraspanin (TSP)-33] (Figure 3). Homologs and/or family members of known schistosome vaccine candidates such as calpain (50) (AY814430), a 28-kDa GST – *Sh*28GST (51) (AY815303), and the TSPs *Sm*-TSP-1 and *Sm*-TSP-2 (33, 52) (AY815196) were also identified. Table 1 lists all of the antigens depicted in Figure 3 along with their *S. haematobium* orthologs as we reasoned that these were probably the native parasite antigens that our DIR and CI sera were targeting during the course of *S. haematobium* infection. Of the 20 antigens that were targets of significantly elevated IgG1 responses in post-treatment DIRs compared to CIs, only 7 (35%) were targets of IgE responses that were deemed to be above the reactivity cut-off (Figure 4).

**Table 1:** **Arrayed proteins significantly reactive to *S. haematobium*-infected DIR post-treatment sera and *S. japonicum*-infected, self-curing rhesus macaque sera**.

Array ID (GenBank accession number)	Description	Reactivity difference^a^ (*p* value)	Frequency of recognition (%)	*S. haematobium* homolog; amino acid identity with arrayed antigen				Therapeutic use
				GenBank accession number	Description/aa homology	Length (aa)	TM domains^b^	
**DIR-reactive proteins**	
AY810792	Butylcholinesterase (*S. japonicum*)	6.97 × 10^−6^	100	MS3_01257	Acetylcholinesterase; 86%	745	1	IgG to *S. mansoni* AchE drives complement-mediated killing of somules by 75–95% (53)
AY812951	Mastin (*S. japonicum*)	7.10 × 10^−6^	90	MS3_04920	Plasminogen; 70%	492	1	
AY815196	Similar to NM_079585 tetraspanin 86D in *Drosophila melanogaster* (*S. japonicum*)	1.40 × 10^−5^	50	MS3_02232	Tetraspanin-33; 81%	259	4	Vaccination with members of this family (*Sm*-TSP-1 and *Sm*-TSP-2) induces 65–69% protection in a mouse model of schistosomiasis (33). Vaccination with a member of this family (Sj23) induces 35% protection in a mouse model of schistosomiasis (54)
AY815945	SJCHGC09124 protein (*S. japonicum*)	2.29 × 10^−5^	100	MS3_10649	Hypothetical protein; 73%	141^c^	3	
AY809911	SJCHGC02149 protein (*S. japonicum*); putative immunoglobulin domain superfamily (sensory guidance protein) (*S. mansoni*); 90%	3.06 × 10^−5^		MS3_07405	Hypothetical protein; 87%	574^c^	1	
C609117.1	Succinate dehydrogenase (*S. mansoni*)	1.37 × 10^−4^	100	MS3_03684	Succinate dehydrogenase cytochrome b560 subunit, mitochondrial; 93%	379	2	
AY815690	Myosin-7 (*S. japonicum*)^d^	2.25 × 10^−4^	80	MS3_09744	Ribosome-binding protein 1; 90%	775	0	
AY812591	SJCHGC04069 protein (*S. japonicum*)	4.25 × 10^−4^	100	MS3_01313	Hypothetical protein (RNA binding); 71%	392	0	
AY222868	SJCHGC06654 protein (*S. japonicum*)	4.45 × 10^−4^	90	MS3_04717	Large subunit ribosomal protein; 48%	150	1	
AY808953	Zinc finger CCCH domain-containing protein 3 (*S. japonicum*)	0.0023	100	MS3_10292	Hypothetical protein; 41%	201	0	
AY814497	SJCHGC02432 protein (*S. japonicum*)	0.0027	100	MS3_04817	Hypothetical protein; 58%	351	0	
AY814261	Ectonucleotide pyrophosphatase/phosphodiesterase family member 5 (*S. japonicum*)	0.0033	90	MS3_08684	Ectonucleotide pyrophosphatase/phosphodiesterase family member 5; 67%	452	1	Suppression of *S. mansoni* ortholog (SmNPP-5) impairs the parasite’s ability to establish infection *in vivo* (55)
AY816000	Cytochrome b-561 (*S. japonicum*)	0.0081	60	MS3_10028	Cytochrome b-561; 85%	242	6	
AY815303	Similar to microsomal glutathione *S*-transferase in *Oryctolagus cuniculus* (*S. japonicum*)	0.0099	50	MS3_02176	Microsomal glutathione *S*-transferase 3; 85%	151	3	A member of this protein family (*Sh*28GST) is undergoing clinical trial as a vaccine against *S. haematobium* (51)
AY810700	Solute carrier family 2 protein (*S. japonicum*)	0.0100	50	MS3_02545	Solute carrier family 2, facilitated glucose transporter member 1; 85%	522	12	Suppression of *S. mansoni* ortholog (SGTP1) impairs the parasite’s ability to establish infection *in vivo* (56)
AY812972	SJCHGC02374 protein (*S. japonicum*)	0.0106	60	MS3_11481	Hypothetical protein; 90%	71^c^	0	
AY814817	SJCHGC06849 protein (*S. japonicum*)	0.0130	90	MS3_05945	Hypothetical protein (TATA-box binding); 71%	416^c^	0	
AY815056	SJCHGC06191 protein (*S. japonicum*), marvel-containing potential lipid-raft-associated protein (*S. mansoni*); 90%	0.0155	80	MS3_07473	Hypothetical protein; 91%	215	4	
AY814977	Nervana 2 (*S. japonicum*)	0.0381	70	MS3_03655	Sodium/potassium-transporting ATPase subunit beta-2; 87%	293	1	Suppression of *S. mansoni* ortholog (SNaK1β) impairs the parasite’s ability to establish infection *in vivo* (57)
AY814430	Calpain (*S. japonicum*)	0.0497	100	MS3_02003	Calpain; 83%	2028	0	*S. mansoni* ortholog (Smp80) induces 64% protection in a baboon model of schistosomiasis (50)
**Macaque-reactive proteins**	
AY815838	SJCHGC05998 protein (*S. japonicum*)	1.18 × 10^−8e^, 2.29 × 10^−8f^	100	N/A				
AY812161	UPF05056 protein (*S. japonicum*)	8.72 × 10^−4 e^, 1.02 × 10^−6f^	100	N/A				
AY815056	SJCHGC06191 protein (*S. japonicum*), marvel-containing potential lipid-raft-associated protein (*S. mansoni*); 90%	5.80 × 10^−8e^, 2.36 × 10^−4f^	100	N/A				
AY810700	Solute carrier family 2 protein (*S. japonicum*)	0.0071^e^	33	N/A				Suppression of *S. mansoni* ortholog (SGTP1) impairs the parasite’s ability to establish infection *in vivo* (56)
AY812195	Extracellular superoxide dismutase (Cu–Zn) (*S. japonicum*)	0.0071^f^	83	N/A				*S. mansoni* ortholog (SmCT-SOD) induces 39% protection in a mouse model of schistosomiasis (58)
AY814158	Major egg antigen (p40) (*S. japonicum*)	0.0444^e^	67	N/A				
AY808379	SJCHGC09517 protein (*S. japonicum*)	0.0454^e^	17	N/A				
AY09526	SJCHGC09219 protein (*S. japonicum*)	0.0504^e^, 0.0067^f^	67	N/A				

*^a^For DIR-reactive proteins, difference is in elevation of IgG1 response of DIRs compared to CIs post-treatment*.

*^b^Transmembrane (TM) domains predicted by TMHMM 2.0. For full-length proteins, first TM domain contains an N-terminal signal sequence*.

*^c^Proteins lack start methionine*.

*^d^We believe the sequence represented by AY815690 [“myosin-7 (*S. japonicum*)”] has been incorrectly annotated due to its high degree of homology with other parasite orthologs of ribosome-binding protein 1 and lack of hits with any form of myosin*.

*^e^Difference in elevation of IgG response between 0 and 12 weeks p.i*.

*^f^Difference in elevation of IgG response between 0 and 20 weeks p.i*.

### Transcription analysis

**Figure 5:**
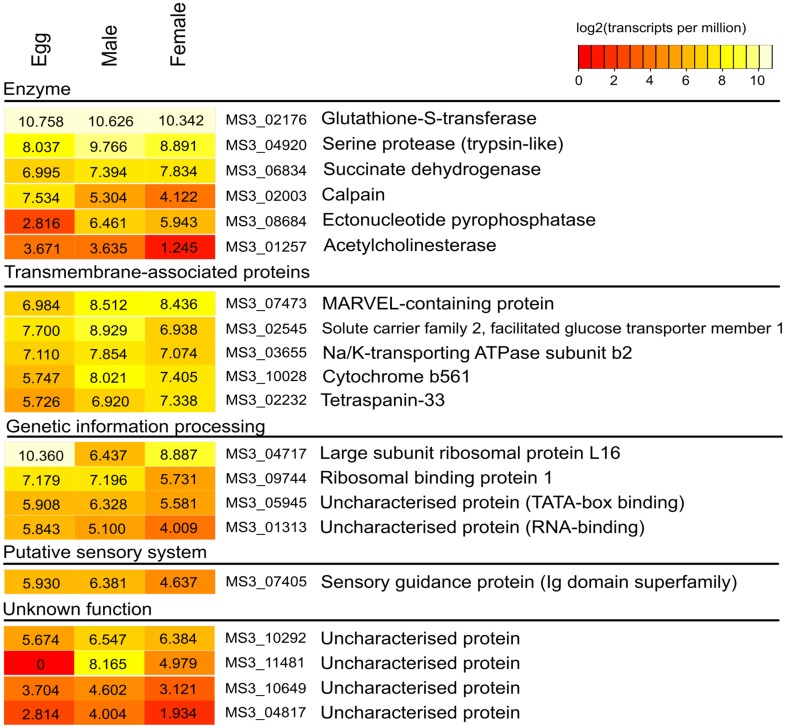
**Gene transcription in the adult and egg stages of *S. haematobium* for all arrayed proteins inducing significantly different and reactive IgG responses to DIR post-treatment sera**. Data were assembled from publicly available RNA-seq databases (43). These data were filtered for quality (PHRED score of >30) using Trimmomatic (8) and aligned to the open reading frames of the published gene set (7) using Bowtie (v2.1.0) (9). Normalized levels of gene transcription were calculated using the software package RSEM (v1.2.11) (10) and reported as the numbers of transcripts per million reads sequenced (TPMs). The TPM value of each gene was log_2_-transformed and subjected to heat map visualization using R.

The transcription of genes in the adult and egg stages of *S. haematobium* was assessed for orthologs of all 20 arrayed *S. mansoni* and *S. japonicum* proteins that were the target of significantly different DIR IgG responses post-treatment using publicly available RNA-seq data. We did not find any significant difference in the level of transcription between life stages for a given protein. MS3_02176 (the gene encoding microsomal GST-3) was expressed most highly and relatively constitutively in all developmental stages examined (Figure 5).

### IgG profiles of *S. japonicum*-infected self-curing rhesus macaques differ during the course of infection

**Figure 6:**
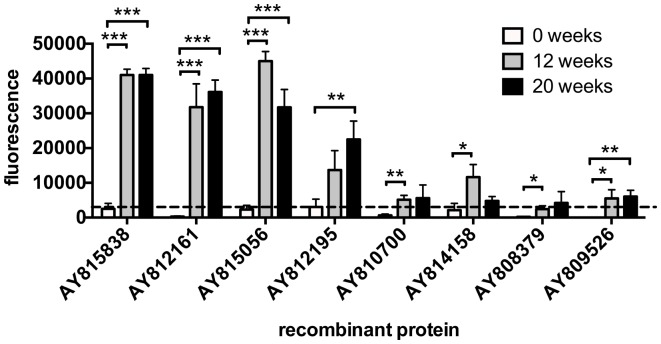
**IgG antibody profiles to arrayed antigens differ in *Schistosoma japonicum*-infected, self-curing rhesus macaques during the course of infection from exposure to perfusion**. Average adjusted signal intensity values depicting IgG antibody responses to each significantly reactive antigen are shown at baseline (0 weeks), 12 weeks post-infection, and elimination (20 weeks post-infection). The dashed line is the cut-off for IgG reactivity (3210), calculated as one standard deviation of the mean of the no-DNA control spots on the array. Statistical analysis was performed using Student’s *t*-test. *p < 0.05, **p < 0.01, ***p < 0.001.

To investigate IgG responses of rhesus macaques to arrayed proteins during the course of a self-curing infection, protein arrays were probed with sera taken at week 0 (primary infection), week 12, and week 20 (after parasite elimination). Antibody responses to all (eight proteins – Table 1) but one reactive protein (AY812195 – extracellular SOD) were significantly elevated between 0 and 12 weeks post-infection (p.i.), with the three most robust and highly significant responses being aimed at proteins of unknown function (AY815838 and AY812161) and a MARVEL domain-containing lipid-raft-associated protein (AY815056). The IgG reactivity of only one protein (AY812195 – extracellular SOD) was elevated at 20 weeks compared to 12 weeks p.i. (Figure 6; Table 1).

### Three different disease models of resistance to schistosomiasis reveal common reactivity to some arrayed proteins

**Figure 7:**
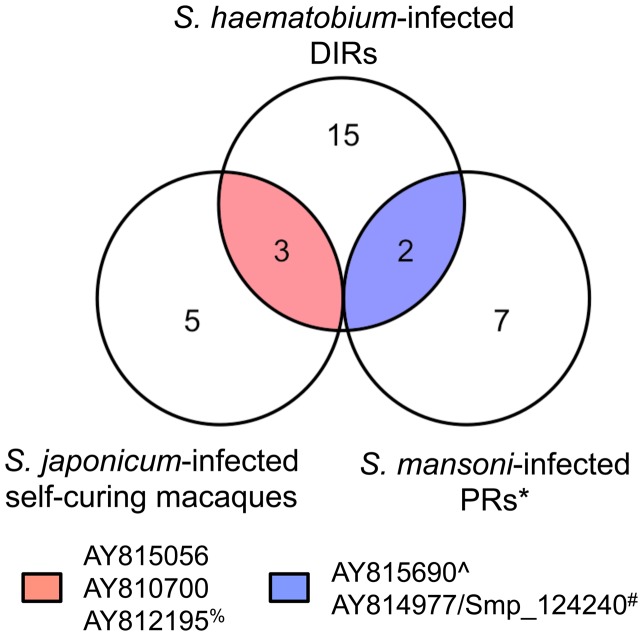
**Different disease models of schistosomiasis resistance show common IgG responses to some arrayed antigens**. Venn diagram depicting common IgG reactive proteins between *Schistosoma haematobium*-infected humans from an endemic area in Africa who acquire drug-induced resistance (DIR) after praziquantel treatment, *Schistosoma japonicum*-infected self-curing rhesus macaques, and *Schistosoma mansoni*-infected humans from an endemic area of Brazil who are naturally resistant (PRs). *Data from Gaze et al. (20); ^%^IgG1 response to AY812195 is significantly different between DIRs before and after praziquantel treatment but not between DIRs and CIs post-treatment; ^#^AY814977 and Smp_124240 are the respective *S. japonicum* and *S. mansoni* orthologs of SNaK1β. ^∧^We believe the sequence represented by AY815690 [“myosin-7” (*S. japonicum*)] has been incorrectly annotated due to its high degree of homology with other parasite orthologs of ribosome-binding protein 1 and lack of BlastP hits with any form of myosin.

We searched for reactive proteins common to DIR human subjects, *S. japonicum*-infected self-curing rhesus macaques (both described herein), and humans living in an *S. mansoni*-endemic area of Brazil who, unlike DIRs, have never been treated with PZQ but are putatively resistant to infection (20). Three reactive proteins were common targets of “protective” antibody responses in the DIR and macaque models: a MARVEL domain-containing lipid-raft-associated protein; a glucose transporter (SGTP1); and an extracellular SOD (although the IgG response to this protein was not significantly elevated between DIRs and CIs after PZQ treatment). Two reactive antigens were commonly recognized by both DIRs and PRs: ribosome-binding protein 1 and the beta subunit of Na^+^/K^+^ ATPase (SNaK1β) (Figure 7).

## Discussion

The critical role that antibodies play in resistance to schistosomiasis resistance has been well established in animal models by numerous passive transfer studies [e.g., Ref. (59, 60)], and there is evidence that some mechanisms of protective immunity in humans are antibody-mediated, both in individuals naturally resistant to schistosomiasis (20) and those who acquire resistance after PZQ therapy (25). Herein, we describe the antibody reactivity profiling of a schistosome protein array with sera from *S. haematobium*-exposed DIR and CI individuals and rhesus macaques self-cured of a *S. japonicum* experimental infection (34) in an effort to identify schistosome antigens that might be the targets of resistant human and non-human primate hosts. We previously utilized this protein microarray to define the antibody signatures of individuals that are either naturally resistant to or CI with *S. mansoni* in a schistosomiasis endemic area of Brazil (20). We restricted our antibody isotype analyses to IgG1 and IgE. IgG1 is one of the main drivers of the protective humoral response to schistosomiasis (23, 24), an observation supported by studies showing that key tegument vaccine antigens like Smp80 (calpain), *Sm*-TSP-2, and Sm29 are the targets of these responses in schistosome-resistant individuals (32, 33, 61). IgE is thought to be critical in resistance to schistosomiasis, including the DIR process (25, 62, 63), but caution is warranted in development of anti-helminth vaccines that drive IgE responses due to potential anaphylactic responses in individuals who are pre-sensitized from chronic helminth infection/exposure (64).

Significantly elevated IgG1 responses were detected to 24 antigens in DIR subjects 18 months after therapy compared to pre-treatment responses. In stark contrast, we did not detect elevated IgG1 responses to any proteins in CI subjects at 18 months post-treatment compared to pre-treatment levels. None of these antigens were recognized in a previous study by us in which pooled sera from *S. haematobium*-exposed individuals before and after PZQ treatment were used to probe 2D gels containing *S. haematobium* SWAP (27), likely because the majority of proteins on the array are membrane-associated tegument proteins and might not be well represented in SWAP due to the very mild solubilizing nature (Tris) of the preparation.

It is noteworthy that IgG1 reactivity to a further 105 (48%) arrayed antigens was significantly higher in post-compared to pre-treatment DIRs but signal intensities were below the cut-off, so the proteins were deemed non-reactive. This decreased level of reactivity possibly reflects the heterogeneity of the antigen–antibody interaction, i.e., antibodies to *S. haematobium* proteins are reacting with a protein array containing *S. mansoni* and *S. japonicum* antigens. Indeed, significant differences in antibody recognition patterns were observed when using sera from *S. haematobium*-exposed people to probe crude antigen preparations from the closely related *S. bovis*, and vice versa (65). Moreover, sequence variation in the epitopes of *Sh*28GST, and its homologs from *S. mansoni* and *S. bovis* significantly altered the immune response generated by the host (66).

Twenty reactive arrayed antigens were the targets of significantly greater IgG1 responses in DIRs compared to CIs post-treatment. A further 72 (33%) proteins were the target of significantly different IgG1 recognition profiles between DIRs and CIs but were below the reactivity cut-off. We hypothesize that at least some of these IgG1-reactive proteins are major targets of protective immunity, engendering resistance to schistosomiasis through an antibody-mediated neutralization of the cognate antigen, the role of which is essential to the survival of the parasite within the host (e.g., nutrient acquisition, immune evasion) such that disruption of its function results in worm impairment. Indeed, some of these antigens are protective in animal challenge models of schistosomiasis; for example, vaccination with and the Ca^2+^-activated protease, calpain (AY814430), induces 64% in baboons (50). *Sh*28GST (a homolog of the arrayed immunoreactive protein AY815303) is a multi-functional enzyme present in the tegument and sub-tegument of adult (67) and larval (68) schistosomes and the current focus of vaccine trials in humans (51). Its exact function is unknown [studies suggest it may aid in immune evasion by the parasite through its role in fatty acid metabolism and prostaglandin D2 synthesis (69), but vaccine efficacy has been attributed to the induction of antibodies that neutralize enzyme activity (70)]. Other extracellular enzymes were prominent amongst the IgG1-reactive proteins, including proteases (calpain, mastin), esterases, and superoxide dismutase, so it is tempting to speculate that antibodies to these enzymes neutralize key physiological processes (71, 72), and this now warrants further investigation. Members of the TSP family in schistosomes (*Sm*-TSP-1 and *Sm*-TSP-2) are four-transmembrane domain proteins located within the tegument of larval and adult worms that have functions in membrane biogenesis (73). TSP-based vaccines have shown to be efficacious against schistosomiasis with *Sm*-TSP-1 and *Sm*-TSP-2 (33, 52) and *Sj*23 (54, 74) conferring protection in animal challenge models.

Other significant IgG1 responses were aimed at tegument-associated proteins that play fundamental roles in parasitism. Surface-associated acetylcholinesterase (AChE) (AY810792) has been implicated in the regulation of glucose scavenging from host blood (75) and anti-AChE antibodies facilitate complement-mediated killing of larval schistosomes (53). Genes encoding the glucose transporter SGTP1 (AY810700), Na^+^/K^+^ ATPase subunit SNaK1β (AY814977) and ectonucleotide pyrophosphatase/phosphodiesterase SmNPP-5 (AY814261) have all been functionally silenced within schistosomes using RNAi (55–57), resulting in impairment of the worm’s ability to establish infection in the host and highlighting their importance to parasite survival.

Significantly IgG1-reactive proteins whose therapeutic potential has not yet been examined include mastin (AY812951) and a MARVEL domain-containing lipid-raft-associated protein (AY815056). Mastin is a trypsin-like serine protease and, in schistosomes, proteases of this class are known as cercarial elastases (CEs) for their role in skin degradation to facilitate penetration of the free-living cercaria into the definitive host (76). Mastin, however, differs in structural homology to CEs and has been assigned to a group of “non-CE” serine proteases (77). The five members of this group are yet to be functionally characterized in terms of their roles in parasitism, but mastin is unique in that it is highly upregulated in the intra-mammalian schistosomula and adult stages [60 and 150% relative to the constitutively expressed *smcox1*, respectively (77) compared to the free-living stages of the parasite (78, 79), alluding to a fundamental parasitic function]. MARVEL domains have four-transmembrane helix architecture and proteins containing these motifs associate with membrane micro-domains and have been implicated in membrane biogenesis (80). In a pathogenesis context, the MARVEL domain-containing protein Nce102 regulates actin organization and invasive growth of *Candida albicans*, with Nce102 deletion mutants showing decreased virulence in mice (81). Antigens such as mastin and the MARVEL domain protein are attractive vaccine candidates for the reasons described herein as well as the successful use of proteases (82–84) and membrane structural proteins as anti-helminth vaccines [e.g., Ref. (33, 54, 85, 86)].

A group of ribosome-associated proteins were also the targets of significantly higher IgG1 responses in DIRs compared to CIs post-treatment and included ribosome-binding protein 1. Ribosome-associated proteins have received attention in the field of parasite immunology because of their classification as “patho-antigens” – conserved intracellular molecules capable of inducing an immunopathological response (87). Patho-antigens such as acidic ribosomal protein P0 conferred protection as vaccines against the intracellular parasites *Leishmania major* (87) and *Plasmodium yoelii* (88) in mouse challenge models of infection, and antibodies to *P. falciparum* P0 have been detected in individuals who are immune to malaria (89). The roles of these antigens, such as ribosome-binding protein 1, in the induction of anti-schistosome immunity is unclear, but it is possible that these intracellular molecules are stimulating host immune effectors through exosome-mediated pathways [recently identified in related helminths (90, 91)]. It should also be noted that ribosome-binding protein 1 was one of the two antigens recognized by both *S. mansoni*-exposed PR subjects in Brazil and *S. haematobium*-exposed DIR subjects in Africa (Figure 6), possibly highlighting a common role in different mechanisms of schistosomiasis resistance.

IgE responses to arrayed antigens were, for the most part, significantly weaker in post-therapy DIRs compared to pre-treatment responses, which appears to be in contrast to the positive association between IgE levels and the process of acquiring DIR status (25, 62). This could be likely for two reasons: (1) these earlier studies on DIR employed soluble antigen preparations to detect IgE responses, whereas the majority of arrayed proteins are membrane-associated and therefore would not have been present in buffer-soluble parasite extracts or (2) the DIR cohort, being egg-negative, does not receive the IgE-inducing stimulus of egg antigens (92). The latter explanation may be supported by the case of extracellular SOD (AY812195); the IgE response to this protein was significantly lower in egg-negative, post-treatment DIRs (Figure 2B) but significantly higher in egg-positive, post-treatment CIs (data not shown). Indeed, a recent study describing the prediction of IgE-binding antigens in *S. mansoni*-infected individuals reported no significant change in the IgE response to extracellular SOD before and 5 weeks after PZQ treatment (93), which lends support to the observation that the waning IgE response to some antigens in DIRs might be due to the reduced amount of IgE-inducing stimulus received by this cohort. Less than half of the antigens that were significantly reactive for DIR post-treatment IgG1 compared to pre-treatment levels were reactive (above the cut-off) for IgE responses.

IgE poses somewhat of a conundrum for helminth vaccinologists due to its clear association with naturally acquired protection (22, 63), but the accompanying risk of vaccinating people with a recombinant protein that is the target of pre-existing IgE responses and poses the risk of inducing atopy (64), or potentially anaphylaxis. Instead of excluding potentially protective IgG1-inducing antigens that are the targets of parasite-derived IgE in exposed individuals from further vaccine development, we propose that the molecules be assessed for allergenicity through the use of basophil-activation studies, given that the induction of IgE and clinical manifestation of allergy are not mutually inclusive events (94). Another strategy aimed at minimizing potential allergenicity of helminth proteins involves their fusion to Fcγ, thereby directing the chimeric protein to the negative signaling receptor FcγRIIb expressed on pro-allergic cells (95).

The IgG1 response in *S. japonicum*-infected self-curing macaques to the majority of reactive antigens was significantly higher at 12 weeks p.i. [around the time that worm death starts to occur (34)] compared to week 0. Proteins that were the target of these antibodies included a protein with weak sequence homology to a bacterial hydrolase (AY815838), extracellular SOD (AY812195), and the previously discussed glucose transport and MARVEL domain-containing proteins. Extracellular SOD is thought to facilitate the parasite’s evasion of the immune response by neutralizing the effects of reactive oxygen and nitrogen species and has proven efficacious in murine vaccine trials (58). Moreover, both hydrolases and anti-oxidant enzymes were suggested to be the targets of IgG-mediated worm elimination in a previously established macaque self-cure model of schistosomiasis (34).

Given the cognate recognition of antigen by both B and helper T cells in the immune response, we hypothesize that the best antigens for a recombinant protein vaccine are those that elicit responses by both antibodies and T cells during the acquisition of DIR. The antigens described herein should now be subjected to further refinement by assessing their ability to drive T-cell proliferation *ex vivo*. T-cell profiling of B-cell antigens has been conducted for the vaccinia virus (discovered using protein array profiling) where plasmids encoding arrayed proteins were expressed as inclusion bodies and screened for T-cell reactivity in a high-throughput format (96).

In this pilot study, we have described the screening of a schistosome protein array to identify potential targets of protective immunity in *S. haematobium*-infected people who acquire DIR after PZQ treatment, with the hypothesis that these antigens are responsible for essential parasitic functions such that antibody-mediated neutralization of these molecules result in worm impairment or death. While the modest number of targets identified from this work may be reflective of the heterogeneity between the antigens and sera used in the study, a benefit of this approach is the identification of proteins that are cross-reactive between *S. haematobium*, *S. japonicum*, and *S. mansoni*, a desirable feature of a vaccine antigen if it is to be protective against all medically important schistosome species. If a pan-schistosome vaccine is developed, it will likely be part of a control program that integrates a vaccination cocktail of multiple recombinant antigens with chemotherapy, and so a comprehensive portfolio of the targets of DIR is a crucial component of the vaccine discovery strategy. Future iterations of our protein array will be expanded to represent even more of the schistosome proteome, ensuring that an extensive complement of DIR-reactive vaccine antigens will be available for progression into further development.

## Conflict of Interest Statement

The authors declare that the research was conducted in the absence of any commercial or financial relationships that could be construed as a potential conflict of interest.

## Footnotes

^1^http://www.R-project.org

^2^http://cran.r-project.org/web/packages/heatmap.plus

## References

[1] Steinmann P Keiser J Bos R Tanner M Utzinger J. Schistosomiasis and water resources development: systematic review, meta-analysis, and estimates of people at risk. Lancet Infect Dis (2006) 6(7):411–25. 10.1016/S1473-3099(06)70521-7 16790382

[2] Butterworth AE Hagan P Immunity in human schistosomiasis. Parasitol Today (1987) 3(1):11–6 10.1016/0169-4758(87)90091-3 15462862

[3] Cheever AW Torky AH Shirbiney M. The relation of worm burden to passage of *Schistosoma haematobium* eggs in the urine of infected patients. Am J Trop Med Hyg (1975) 24(2):284–8. 1119670 10.4269/ajtmh.1975.24.284

[4] Kjetland EF Leutscher PD Ndhlovu PD A review of female genital schistosomiasis. Trends Parasitol (2012) 28(2):58–65 10.1016/j.pt.2011.10.008 22245065

[5] Moller H Heseltine E Vainio H. Working group report on schistosomes, liver flukes and Helicobacter pylori. Int J Cancer (1995) 60(5):587–9. 10.1002/ijc.2910600502 7860130

[6] Secor WE. The effects of schistosomiasis on HIV/AIDS infection, progression and transmission. Curr Opin HIV AIDS (2012) 7(3):254–9. 10.1097/COH.0b013e328351b9e3 22327410 PMC11316515

[7] Harnett W The anthelmintic action of praziquantel. Parasitol Today (1988) 4(5):144–6 10.1016/0169-4758(88)90192-5 15463071

[8] Harder A Andrews P Thomas H Praziquantel: mode of action. Biochem Soc Trans (1987) 15(1):68–70. 3493930 10.1042/bst0150068

[9] Ottesen EA Hiatt RA Cheever AW Sotomayor ZR Neva FA. The acquisition and loss of antigen-specific cellular immune responsiveness in acute and chronic schistosomiasis in man. Clin Exp Immunol (1978) 33(1):37–47. 709912 PMC1537513

[10] Grogan JL Kremsner PG van Dam GJ Metzger W Mordmuller B Deelder AM Antischistosome IgG4 and IgE responses are affected differentially by chemotherapy in children versus adults. J Infect Dis (1996) 173(5):1242–7. 10.1093/infdis/173.5.1242 8627078

[11] Grzych JM Grezel D Xu CB Neyrinck JL Capron M Ouma JH IgA antibodies to a protective antigen in human schistosomiasis mansoni. J Immunol (1993) 150(2):527–35. 8419485

[12] Mutapi F. Heterogeneities in anti-schistosome humoral responses following chemotherapy. Trends Parasitol (2001) 17(11):518–24. 10.1016/S1471-4922(01)02118-3 11872396

[13] Mutapi F Ndhlovu PD Hagan P Spicer JT Mduluza T Turner CM Chemotherapy accelerates the development of acquired immune responses to *Schistosoma haematobium* infection. J Infect Dis (1998) 178(1):289–93. 10.1086/517456 9652458

[14] Bourke CD Nausch N Rujeni N Appleby LJ Mitchell KM Midzi N Integrated analysis of innate, Th1, Th2, Th17, and regulatory cytokines identifies changes in immune polarisation following treatment of human schistosomiasis. J Infect Dis (2013) 208(1):159–69. 10.1093/infdis/jis524 23045617 PMC3666130

[15] Mduluza T Mutapi F Ruwona T Kaluka D Midzi N Ndhlovu PD. Similar cellular responses after treatment with either praziquantel or oxamniquine in *Schistosoma mansoni* infection. Malawi Med J (2009) 21(4):176–82. 10.4314/mmj.v21i4.49642 21174933 PMC3345748

[16] Milner T Reilly L Nausch N Midzi N Mduluza T Maizels R Circulating cytokine levels and antibody responses to human *Schistosoma haematobium*: IL-5 and IL-10 levels depend upon age and infection status. Parasite Immunol (2010) 32(11–12):710–21. 10.1111/j.1365-3024.2010.01235.x 21039611 PMC3033519

[17] Dunne DW Butterworth AE Fulford AJ Kariuki HC Langley JG Ouma JH Immunity after treatment of human schistosomiasis: association between IgE antibodies to adult worm antigens and resistance to reinfection. Eur J Immunol (1992) 22(6):1483–94. 10.1002/eji.1830220622 1601036

[18] Naus CW van Dam GJ Kremsner PG Krijger FW Deelder AM. Human IgE, IgG subclass, and IgM responses to worm and egg antigens in schistosomiasis haematobium: a 12-month study of reinfection in Cameroonian children. Clin Infect Dis (1998) 26(5):1142–7. 10.1086/520310 9597243

[19] Satti MZ Lind P Vennervald BJ Sulaiman SM Daffalla AA Ghalib HW. Specific immunoglobulin measurements related to exposure and resistance to *Schistosoma mansoni* infection in Sudanese canal cleaners. Clin Exp Immunol (1996) 106(1):45–54. 10.1046/j.1365-2249.1996.d01-810.x 8870697 PMC2200570

[20] Gaze S Driguez P Pearson MS Mendes T Doolan DL Trieu A An immunomics approach to schistosome antigen discovery: antibody signatures of naturally resistant and chronically infected individuals from endemic areas. PLoS Pathog (2014) 10(3):e1004033. 10.1371/journal.ppat.1004033 24675823 PMC3968167

[21] Sabah AA Fletcher C Webbe G Doenhoff MJ. *Schistosoma mansoni*: chemotherapy of infections of different ages. Exp Parasitol (1986) 61(3):294–303. 10.1016/0014-4894(86)90184-0 3086114

[22] Correa-Oliveira R Caldas IR Gazzinelli G. Natural versus drug-induced resistance in *Schistosoma mansoni* infection. Parasitol Today (2000) 16(9):397–9. 10.1016/S0169-4758(00)01740-3 10951600

[23] Mitchell KM Mutapi F Savill NJ Woolhouse ME. Protective immunity to *Schistosoma haematobium* infection is primarily an anti-fecundity response stimulated by the death of adult worms. Proc Natl Acad Sci U S A (2012) 109(33):13347–52. 10.1073/pnas.1121051109 22847410 PMC3421178

[24] Wilson S Jones FM van Dam GJ Corstjens PL Riveau G Fitzsimmons CM Human *Schistosoma haematobium* antifecundity immunity is dependent on transmission intensity and associated with immunoglobulin G1 to worm-derived antigens. J Infect Dis (2014) 210(12):2009–16. 10.1093/infdis/jiu374 25001462 PMC4241947

[25] Black CL Mwinzi PN Muok EM Abudho B Fitzsimmons CM Dunne DW Influence of exposure history on the immunology and development of resistance to human schistosomiasis mansoni. PLoS Negl Trop Dis (2010) 4(3):e637. 10.1371/journal.pntd.0000637 20351784 PMC2843635

[26] Black CL Steinauer ML Mwinzi PN Evan Secor W Karanja DM Colley DG. Impact of intense, longitudinal retreatment with praziquantel on cure rates of schistosomiasis mansoni in a cohort of occupationally exposed adults in western Kenya. Trop Med Int Health (2009) 14(4):450–7. 10.1111/j.1365-3156.2009.02234.x 19222824 PMC2941893

[27] Mutapi F Burchmore R Mduluza T Foucher A Harcus Y Nicoll G Praziquantel treatment of individuals exposed to *Schistosoma haematobium* enhances serological recognition of defined parasite antigens. J Infect Dis (2005) 192(6):1108–18. 10.1086/432553 16107967

[28] Mutapi F Burchmore R Mduluza T Midzi N Turner CM Maizels RM. Age-related and infection intensity-related shifts in antibody recognition of defined protein antigens in a schistosome-exposed population. J Infect Dis (2008) 198(2):167–75. 10.1086/589511 18549316

[29] Braschi S Curwen RS Ashton PD Verjovski-Almeida S Wilson A. The tegument surface membranes of the human blood parasite *Schistosoma mansoni*: a proteomic analysis after differential extraction. Proteomics (2006) 6(5):1471–82. 10.1002/pmic.200500368 16447162

[30] Braschi S Wilson RA. Proteins exposed at the adult schistosome surface revealed by biotinylation. Mol Cell Proteomics (2006) 5(2):347–56. 10.1074/mcp.M500287-MCP200 16269422

[31] Cardoso FC Macedo GC Gava E Kitten GT Mati VL de Melo AL *Schistosoma mansoni* tegument protein Sm29 is able to induce a Th1-type of immune response and protection against parasite infection. PLoS Negl Trop Dis (2008) 2(10):e308. 10.1371/journal.pntd.0000308 18827884 PMC2553283

[32] Cardoso FC Pacifico RN Mortara RA Oliveira SC. Human antibody responses of patients living in endemic areas for schistosomiasis to the tegumental protein Sm29 identified through genomic studies. Clin Exp Immunol (2006) 144(3):382–91. 10.1111/j.1365-2249.2006.03081.x 16734606 PMC1941986

[33] Tran MH Pearson MS Bethony JM Smyth DJ Jones MK Duke M Tetraspanins on the surface of *Schistosoma mansoni* are protective antigens against schistosomiasis. Nat Med (2006) 12(7):835–40. 10.1038/nm1430 16783371

[34] Wilson RA Langermans JA van Dam GJ Vervenne RA Hall SL Borges WC Elimination of *Schistosoma mansoni* adult worms by rhesus macaques: basis for a therapeutic vaccine? PLoS Negl Trop Dis (2008) 2(9):e290. 10.1371/journal.pntd.0000290 18820739 PMC2553480

[35] Cheever AW Powers KG *Schistosoma mansoni* infection in rhesus monkeys: changes in egg production and egg distribution in prolonged infections in intact and splenectomized monkeys. Ann Trop Med Parasitol (1969) 63(1):83–93. 4980588 10.1080/00034983.1969.11686603

[36] McMullen DB Ritchie LS Oliver-Gonzalez J Knight WB *Schistosoma mansoni* in *Macaca mulatta*. Long-term studies on the course of primary and challenge infections. Am J Trop Med Hyg (1967) 16(5):620–7. 4964092

[37] Driguez P Doolan DL Molina DM Loukas A Trieu A Felgner PL Protein microarrays for parasite antigen discovery. Methods Mol Biol (2015) 1201:221–33. 10.1007/978-1-4939-1438-8_13 25388117

[38] Crompton PD Kayala MA Traore B Kayentao K Ongoiba A Weiss GE A prospective analysis of the Ab response to *Plasmodium falciparum* before and after a malaria season by protein microarray. Proc Natl Acad Sci U S A (2010) 107(15):6958–63. 10.1073/pnas.1001323107 20351286 PMC2872454

[39] Doolan DL Mu Y Unal B Sundaresh S Hirst S Valdez C Profiling humoral immune responses to *P. falciparum* infection with protein microarrays. Proteomics (2008) 8(22):4680–94. 10.1002/pmic.200800194 18937256 PMC3021802

[40] Trieu A Kayala MA Burk C Molina DM Freilich DA Richie TL Sterile protective immunity to malaria is associated with a panel of novel *P. falciparum* antigens. Mol Cell Proteomics (2011) 10(9):M111007948. 10.1074/mcp.M111.007948 21628511 PMC3186199

[41] Driguez P Doolan DL Loukas A Felgner PL McManus DP Schistosomiasis vaccine discovery using immunomics. Parasit Vectors (2010) 3:4 10.1186/1756-3305-3-4 20181031 PMC2837634

[42] McWilliam HE Driguez P Piedrafita D McManus DP Meeusen EN. Discovery of novel *Schistosoma japonicum* antigens using a targeted protein microarray approach. Parasit Vectors (2014) 7:290. 10.1186/1756-3305-7-290 24964958 PMC4080988

[43] Young ND Jex AR Li B Liu S Yang L Xiong Z Whole-genome sequence of *Schistosoma haematobium*. Nat Genet (2012) 44(2):221–5 10.1038/ng.1065 22246508

[44] Bourke CD Nausch N Rujeni N Appleby LJ Trottein F Midzi N Cytokine responses to the anti-schistosome vaccine candidate antigen glutathione-S-transferase vary with host age and are boosted by praziquantel treatment. PLoS Negl Trop Dis (2014) 8(5):e2846. 10.1371/journal.pntd.0002846 24810615 PMC4014416

[45] Eberl M al-Sherbiny M Hagan P Ljubojevic S Thomas AW Wilson RA. A novel and sensitive method to monitor helminth infections by faecal sampling. Acta Trop (2002) 83(2):183–7. 10.1016/S0001-706X(02)00089-X 12088860

[46] Cai YC Xu JF Steinmann P Chen SH Chu YH Tian LG Field comparison of circulating antibody assays versus circulating antigen assays for the detection of schistosomiasis japonica in endemic areas of China. Parasit Vectors (2014) 7:138. 10.1186/1756-3305-7-138 24684924 PMC3978087

[47] Bolger AM Lohse M Usadel B. Trimmomatic: a flexible trimmer for Illumina sequence data. Bioinformatics (2014) 30(15):2114–20. 10.1093/bioinformatics/btu170 24695404 PMC4103590

[48] Langmead B Salzberg SL. Fast gapped-read alignment with Bowtie 2. Nat Methods (2012) 9(4):357–9. 10.1038/nmeth.1923 22388286 PMC3322381

[49] Li B Dewey CN RSEM: accurate transcript quantification from RNA-Seq data with or without a reference genome. BMC Bioinformatics (2011) 12:323 10.1186/1471-2105-12-323 21816040 PMC3163565

[50] Karmakar S Zhang W Ahmad G Torben W Alam MU Le L Use of an Sm-p80-based therapeutic vaccine to kill established adult schistosome parasites in chronically infected baboons. J Infect Dis (2014) 209(12):1929–40. 10.1093/infdis/jiu031 24436452 PMC4038147

[51] Riveau G Deplanque D Remoue F Schacht AM Vodougnon H Capron M Safety and immunogenicity of rSh28GST antigen in humans: phase 1 randomized clinical study of a vaccine candidate against urinary schistosomiasis. PLoS Negl Trop Dis (2012) 6(7):e1704. 10.1371/journal.pntd.0001704 22802974 PMC3389022

[52] Pearson MS Pickering DA McSorley HJ Bethony JM Tribolet L Dougall AM Enhanced protective efficacy of a chimeric form of the schistosomiasis vaccine antigen Sm-TSP-2. PLoS Negl Trop Dis (2012) 6(3):e1564. 10.1371/journal.pntd.0001564 22428079 PMC3302818

[53] Arnon R Espinoza-Ortega B Tarrab-Hazdai R Acetylcholinesterase of *Schistosoma mansoni* – an antigen of functional implications. Mem Inst Oswaldo Cruz (1987) 82(Suppl 4):163–70 10.1590/S0074-02761987000800028 3509181

[54] Zhu Y Ren J Harn DA Si J Yu C Ming X Protective immunity induced with 23 kDa membrane protein dna vaccine of *Schistosoma japonicum* Chinese strain in infected C57BL/6 mice. Southeast Asian J Trop Med Public Health (2003) 34(4):697–701. 15115073

[55] Bhardwaj R Krautz-Peterson G Da’dara A Tzipori S Skelly PJ. Tegumental phosphodiesterase SmNPP-5 is a virulence factor for schistosomes. Infect Immun (2011) 79(10):4276–84. 10.1128/IAI.05431-11 21825060 PMC3187234

[56] Krautz-Peterson G Simoes M Faghiri Z Ndegwa D Oliveira G Shoemaker CB Suppressing glucose transporter gene expression in schistosomes impairs parasite feeding and decreases survival in the mammalian host. PLoS Pathog (2010) 6(6):e1000932. 10.1371/journal.ppat.1000932 20532163 PMC2880588

[57] Da’dara AA Faghiri Z Krautz-Peterson G Bhardwaj R Skelly PJ. Schistosome Na, K-ATPase as a therapeutic target. Trans R Soc Trop Med Hyg (2013) 107(2):74–82. 10.1093/trstmh/trs020 23222953

[58] Cook RM Carvalho-Queiroz C Wilding G LoVerde PT. Nucleic acid vaccination with *Schistosoma mansoni* antioxidant enzyme cytosolic superoxide dismutase and the structural protein filamin confers protection against the adult worm stage. Infect Immun (2004) 72(10):6112–24. 10.1128/IAI.72.10.6112-6124.2004 15385516 PMC517585

[59] Barker RH Jr Srivastava BS Suri P Goldberg M Knopf PM. Immunoprecipitation analysis of radiolabelled protein antigens biosynthesized in vitro by *S. mansoni*. I. Identification of antigens uniquely recognized by protective antibodies. J Immunol (1985) 134(2):1192–201. 3965570

[60] Moloney NA Webbe G. Antibody is responsible for the passive transfer of immunity to mice from rabbits, rats or mice vaccinated with attenuated *Schistosoma japonicum* cercariae. Parasitology (1990) 100(Pt 2):235–9. 10.1017/S0031182000061230 2111906

[61] Ahmad G Zhang W Torben W Ahrorov A Damian RT Wolf RF Preclinical prophylactic efficacy testing of Sm-p80-based vaccine in a nonhuman primate model of *Schistosoma mansoni* infection and immunoglobulin G and E responses to Sm-p80 in human serum samples from an area where schistosomiasis is endemic. J Infect Dis (2011) 204(9):1437–49. 10.1093/infdis/jir545 21921206 PMC3182311

[62] Fitzsimmons CM Jones FM Pinot de Moira A Protasio AV Khalife J Dickinson HA Progressive cross-reactivity in IgE responses: an explanation for the slow development of human immunity to schistosomiasis? Infect Immun (2012) 80(12):4264–70. 10.1128/IAI.00641-12 23006852 PMC3497412

[63] Hagan P Blumenthal UJ Dunn D Simpson AJ Wilkins HA. Human IgE, IgG4 and resistance to reinfection with *Schistosoma haematobium*. Nature (1991) 349(6306):243–5. 10.1038/349243a0 1898985

[64] Diemert DJ Pinto AG Freire J Jariwala A Hamilton R Periago MV Generalized urticaria induced by the Na-ASP-2 hookworm vaccine – implications for the development of vaccines against helminths. J Allerg Clin Immunol (2012) 130(1):169–76.e6. 10.1016/j.jaci.2012.04.027 22633322

[65] Higon M Cowan G Nausch N Cavanagh D Oleaga A Toledo R Screening trematodes for novel intervention targets: a proteomic and immunological comparison of *Schistosoma haematobium*, *Schistosoma bovis* and *Echinostoma caproni*. Parasitology (2011) 138(12):1607–19. 10.1017/S0031182011000412 21729355 PMC3179331

[66] Trottein F Godin C Pierce RJ Sellin B Taylor MG Gorillot I Inter-species variation of schistosome 28-kDa glutathione S-transferases. Mol Biochem Parasitol (1992) 54(1):63–72. 10.1016/0166-6851(92)90095-2 1518533

[67] Taylor JB Vidal A Torpier G Meyer DJ Roitsch C Balloul JM The glutathione transferase activity and tissue distribution of a cloned Mr28K protective antigen of *Schistosoma mansoni*. EMBO J (1988) 7(2):465–72. 3284744 10.1002/j.1460-2075.1988.tb02834.x PMC454343

[68] Balloul JM Pierce RJ Grzych JM Capron A. In vitro synthesis of a 28 kilodalton antigen present on the surface of the schistosomulum of *Schistosoma mansoni*. Mol Biochem Parasitol (1985) 17(1):105–14. 10.1016/0166-6851(85)90131-8 2414656

[69] Johnson KA Angelucci F Bellelli A Herve M Fontaine J Tsernoglou D Crystal structure of the 28 kDa glutathione S-transferase from *Schistosoma haematobium*. Biochemistry (2003) 42(34):10084–94. 10.1021/bi034449r 12939136

[70] Remoue F Rogerie F Gallissot MC Guyatt HL Neyrinck JL Diakkhate MM Sex-dependent neutralizing humoral response to *Schistosoma mansoni* 28GST antigen in infected human populations. J Infect Dis (2000) 181(5):1855–9. 10.1086/315454 10823801

[71] Dougall AM Skwarczynski M Khoshnejad M Chandrudu S Daly NL Toth I Lipid core peptide targeting the cathepsin D hemoglobinase of *Schistosoma mansoni* as a component of a schistosomiasis vaccine. Hum Vaccin Immunother (2014) 10(2):399–409. 10.4161/hv.27057 24231271 PMC4185900

[72] Hotez PJ Diemert D Bacon KM Beaumier C Bethony JM Bottazzi ME The human hookworm vaccine. Vaccine (2013) 31(Suppl 2):B227–32 10.1016/j.vaccine.2012.11.034 23598487 PMC3988917

[73] Tran MH Freitas TC Cooper L Gaze S Gatton ML Jones MK Suppression of mRNAs encoding tegument tetraspanins from *Schistosoma mansoni* results in impaired tegument turnover. PLoS Pathog (2010) 6(4):e1000840. 10.1371/journal.ppat.1000840 20419145 PMC2855321

[74] Shi F Zhang Y Ye P Lin J Cai Y Shen W Laboratory and field evaluation of *Schistosoma japonicum* DNA vaccines in sheep and water buffalo in China. Vaccine (2001) 20(3–4):462–7. 10.1016/S0264-410X(01)00340-1 11672910

[75] Jones AK Bentley GN Oliveros Parra WG Agnew A. Molecular characterization of an acetylcholinesterase implicated in the regulation of glucose scavenging by the parasite *Schistosoma*. FASEB J (2002) 16(3):441–3. 10.1096/fj.01-0683fje 11821256

[76] Kasny M Mikes L Hampl V Dvorak J Caffrey CR Dalton JP Chapter 4. Peptidases of trematodes. Adv Parasitol (2009) 69:205–97. 10.1016/S0065-308X(09)69004-7 19622410

[77] Horn M Fajtova P Rojo Arreola L Ulrychova L Bartosova-Sojkova P Franta Z Trypsin- and chymotrypsin-like serine proteases in *Schistosoma mansoni* – ‘the undiscovered country’. PLoS Negl Trop Dis (2014) 8(3):e2766. 10.1371/journal.pntd.0002766 24676141 PMC3967958

[78] Gobert GN Tran MH Moertel L Mulvenna J Jones MK McManus DP Transcriptional changes in *Schistosoma mansoni* during early schistosomula development and in the presence of erythrocytes. PLoS Negl Trop Dis (2010) 4(2):e600. 10.1371/journal.pntd.0000600 20161728 PMC2817720

[79] Protasio AV Dunne DW Berriman M. Comparative study of transcriptome profiles of mechanical- and skin-transformed *Schistosoma mansoni* schistosomula. PLoS Negl Trop Dis (2013) 7(3):e2091. 10.1371/journal.pntd.0002091 23516644 PMC3597483

[80] Sanchez-Pulido L Martin-Belmonte F Valencia A Alonso MA MARVEL: a conserved domain involved in membrane apposition events. Trends Biochem Sci (2002) 27(12):599–601 10.1016/S0968-0004(02)02229-6 12468223

[81] Douglas LM Wang HX Konopka JB. The MARVEL domain protein Nce102 regulates actin organization and invasive growth of *Candida albicans*. mBio (2013) 4(6):e723–713. 10.1128/mBio.00723-13 24281718 PMC3870249

[82] El Ridi R Tallima H Selim S Donnelly S Cotton S Gonzales Santana B Cysteine peptidases as schistosomiasis vaccines with inbuilt adjuvanticity. PLoS One (2014) 9(1):e85401. 10.1371/journal.pone.0085401 24465551 PMC3897446

[83] Knox DP Redmond DL Newlands GF Skuce PJ Pettit D Smith WD. The nature and prospects for gut membrane proteins as vaccine candidates for *Haemonchus contortus* and other ruminant trichostrongyloids. Int J Parasitol (2003) 33(11):1129–37. 10.1016/S0020-7519(03)00167-X 13678629

[84] Pearson MS Ranjit N Loukas A. Blunting the knife: development of vaccines targeting digestive proteases of blood-feeding helminth parasites. Biol Chem (2010) 391(8):901–11. 10.1515/BC.2010.074 20482309

[85] Dakshinamoorthy G Munirathinam G Stoicescu K Reddy MV Kalyanasundaram R. Large extracellular loop of tetraspanin as a potential vaccine candidate for filariasis. PLoS One (2013) 8(10):e77394. 10.1371/journal.pone.0077394 24146990 PMC3795629

[86] Dang Z Yagi K Oku Y Kouguchi H Kajino K Matsumoto J A pilot study on developing mucosal vaccine against alveolar echinococcosis (AE) using recombinant tetraspanin 3: vaccine efficacy and immunology. PLoS Negl Trop Dis (2012) 6(3):e1570. 10.1371/journal.pntd.0001570 22479658 PMC3313938

[87] Iborra S Soto M Carrion J Nieto A Fernandez E Alonso C The *Leishmania infantum* acidic ribosomal protein P0 administered as a DNA vaccine confers protective immunity to *Leishmania major* infection in BALB/c mice. Infect Immun (2003) 71(11):6562–72. 10.1128/IAI.71.11.6562-6572.2003 14573678 PMC219595

[88] Chatterjee S Singh S Sohoni R Singh NJ Vaidya A Long C Antibodies against ribosomal phosphoprotein P0 of *Plasmodium falciparum* protect mice against challenge with *Plasmodium yoelii*. Infect Immun (2000) 68(7):4312–8. 10.1128/IAI.68.7.4312-4318.2000 10858250 PMC101754

[89] Chatterjee S Singh S Sohoni R Kattige V Deshpande C Chiplunkar S Characterization of domains of the phosphoriboprotein P0 of *Plasmodium falciparum*. Mol Biochem Parasitol (2000) 107(2):143–54. 10.1016/S0166-6851(99)00226-1 10779592

[90] Buck AH Coakley G Simbari F McSorley HJ Quintana JF Le Bihan T Exosomes secreted by nematode parasites transfer small RNAs to mammalian cells and modulate innate immunity. Nat Commun (2014) 5:5488. 10.1038/ncomms6488 25421927 PMC4263141

[91] Marcilla A Trelis M Cortes A Sotillo J Cantalapiedra F Minguez MT Extracellular vesicles from parasitic helminths contain specific excretory/secretory proteins and are internalized in intestinal host cells. PLoS One (2012) 7(9):e45974. 10.1371/journal.pone.0045974 23029346 PMC3454434

[92] Pearce EJ MacDonald AS The immunobiology of schistosomiasis. Nat Rev Immunol (2002) 2(7):499–511 10.1038/nri843 12094224

[93] Farnell EJ Tyagi N Ryan S Chalmers IW Pinot de Moira A Jones FM Known allergen structures predict *Schistosoma mansoni* IgE-binding antigens in human infection. Front Immunol (2015) 6:26. 10.3389/fimmu.2015.00026 25691884 PMC4315118

[94] Wan D Ludolf F Alanine DG Stretton O Ali Ali E Al-Barwary N Use of humanised rat basophilic leukaemia cell line RS-ATL8 for the assessment of allergenicity of *Schistosoma mansoni* proteins. PLoS Negl Trop Dis (2014) 8(9):e3124. 10.1371/journal.pntd.0003124 25254513 PMC4177753

[95] Zhan B Santiago H Keegan B Gillespie P Xue J Bethony J Fusion of Na-ASP-2 with human immunoglobulin Fcgamma abrogates histamine release from basophils sensitized with anti-Na-ASP-2 IgE. Parasite Immunol (2012) 34(8–9):404–11. 10.1111/j.1365-3024.2012.01371.x 22651670

[96] Davies DH Chun S Hermanson G Tucker JA Jain A Nakajima R T cell antigen discovery using soluble vaccinia proteome reveals recognition of antigens with both virion and nonvirion association. J Immunol (2014) 193(4):1812–27. 10.4049/jimmunol.1400663 25024392 PMC4119580

